# Intergenerational Transmission of Peer Aggression

**DOI:** 10.1007/s10964-022-01638-w

**Published:** 2022-06-03

**Authors:** Maria Wiertsema, Charlotte Vrijen, Rozemarijn van der Ploeg, Tina Kretschmer

**Affiliations:** Faculty of Behavioural and Social Sciences, University of Groningen, Grote Rozenstraat 38, 9712 TJ Groningen, The Netherlands

**Keywords:** Intergenerational transmission, Peer aggression, Harsh parenting, Developmental period, Multiple-generation study

## Abstract

It is plausible that peer aggression—like general forms of aggression—is transmitted from one generation to the next. As such, parental behavior in childhood and adolescence may be associated with offspring aggressive behavior against peers. This study used 1970 British Cohort Study data to test intergenerational transmission of peer aggression. The baseline sample consisted of 13,135 participants. At the first assessment that was used in this study, participants were on average 4.95 years old (*SD* = 0.79; 48.20% female). At the last assessment, participants were on average 33.88 years old (*SD* = 0.36; 52.1% female). Models were computed for early and middle childhood, and adolescence. Significant associations between parents’ and offspring peer aggression were found in most models – especially when correlating aggression in similar developmental periods for parents and children. Other transmission mechanisms such as genetic transmission may be relevant and should be taken into account in future studies.

## Introduction

Children and adolescents who aggress against their peers, i.e., display behaviors with the intention to physically or psychologically hurt or harm others (Berkowitz, 1993), are at greater risk for psychosocial problems such as depression (e.g., Wang et al., 2018) and academic as well as interpersonal difficulties (e.g., Fite et al., 2016; Hubbard et al., 2010). Child and adolescent peer aggression is also associated with higher risk of aggressive behavior later in life (e.g., Fite et al., 2016), for example, toward one’s romantic partner and offspring. It remains unclear, however, whether peer aggression is transmitted to the next generation, and, if so, how. From the perspective of offspring, research into the origins of behavioral problems such as aggressive behavior toward peers has not yet focused on the developmental histories of parents. This is unfortunate as knowledge about intergenerational transmission would be crucial to understand and prevent domino chains of peer problem behavior within families. In other words, by knowing parents’ developmental histories better, maladaptive behavioral development in offspring might be prevented. The present study addresses this gap by examining whether peer aggression is transmitted from parents to offspring and by testing different mechanisms that might explain continuity across generations.

### Transmission of Aggressive Behavior Across Generations

Research on the transmission of general aggression and on related constructs (Kim et al., 2009) is prolific and suggests that child maltreatment (e.g., Armfield et al., 2021), intimate partner violence (Shakoor et al., 2020), delinquent behavior (Thornberry et al., 2003), and conduct problems (Smith & Farrington, 2004) show intergenerational continuity. A meta-analysis on the intergenerational transmission of criminal behavior concluded that offspring of criminal parents were more than twice as likely to also engage in crime compared to offspring of non-criminal parents, with just a slight reduction in effect size when covariates were taken into account (Besemer et al., 2017).

In contrast to this body of literature on transmission of general aggression and related constructs, surprisingly little is known about intergenerational patterns of peer aggression as specific type of aggressive behavior. Although general aggression is a much broader concept than peer aggression and intergenerational continuity may be more easily detected than for a specific concept like peer aggression, it seems plausible that peer aggression is also transmitted to the next generation. Unfortunately, there are hardly any data to rigorously model intergenerational transmission of peer experiences. That is, multiple-generation data with peer aggression assessments in similar developmental periods are needed and this means essentially that children need to be followed into adulthood and their offspring need to be included in the study as well. Moreover, the peer aggression and transmission literature are two rather distinct fields with not very much overlap, which probably also contributes to a lack of recent studies on this topic. In fact, to the knowledge of the authors, only two studies have examined transmission of peer-related aggression but have used high-risk rather than population samples and arrived at different conclusions: Whereas fathers’ peer bullying perpetration was transmitted to offspring (Farrington, 1993), no evidence was found for intergenerational transmission of fathers’ peer teasing (Kerr et al., 2018). By studying this association in a population sample and not solely for fathers, the field gains a better understanding both of long-term correlates of peer aggression as well as on preconception predictors of behavioral development for offspring.

### Mechanisms of Transmission

How would intergenerational transmission of peer aggression work? Parenting is an obvious candidate mechanism as it provides the context in which rules and norms that guide behavior are transmitted from one generation to the next (Serbin & Karp, 2003). In other words, parenting is an expression of parents’ behavioral norms. These might lean toward approval of aggression, which would then be expressed as elevated peer aggression in childhood and adolescence and harsh parenting in adulthood. Offspring of harsh parents are thus exposed to behavioral norms in favor of aggression through parenting, even if they have never directly observed their parents’ behavior toward peers in childhood and adolescence. A related possible mechanism is social learning, whereby parental aggression, for example against a partner or as reflected in harsh parenting, could serve as a model for offspring to observe and learn and can signal that aggressive behavior is an appropriate way to handle conflict and to gain and maintain control in relationships (Ingram et al., 2020). Such a home environment may, in turn, promote aggressive offspring behavior, similarly to it increasing the risk for antisocial and delinquent in offspring (Conger et al., 2003; Dogan et al., 2007).

In statistical terms, for harsh parenting to mediate the link between parent and offspring peer aggression, harsh parenting needs to be a correlate of own earlier peer aggression and be associated with offspring peer aggression. The latter has been shown in a plethora of studies, no matter whether offspring behavior was assessed in early (e.g., Stover et al., 2016) or middle childhood (e.g., Braza et al., 2015), or in adolescence (Yu & Gamble, 2008).

To what extent is harsh parenting a correlate of parents’ own earlier peer aggression, however? Given the relative stability of aggressive behavior across the lifespan (e.g., Huesmann et al., 2009), it is likely that parents’ norms toward aggressive behavior have been shaped earlier in development. In other words, harsh parents will often have been aggressive already earlier in development but then with peers rather than offspring as target. Vice versa, child and adolescent peer aggressors may turn into aggression-promoting parents who engage in harsh parenting practices (Dubow et al., 2003). Although studies on child and adolescent behavioral development as determinant of harsh parenting are scarce, work from neighboring fields suggests, for instance, that irritability – a personality facet associated with aggressive behavior in childhood and adolescence (Leibenluft & Stoddard, 2013) – is linked to harsh parenting (Thartori et al., 2019). Similarly, emotion regulation difficulties, especially impulsivity, is an important correlate of child and adolescent aggression (Bresin, 2019) and is also associated with risk for abusive behavior toward offspring (Miragoli et al., 2020). This means that personality traits, and maybe also genetic predisposition for aggressive behavior (Tuvblad & Baker, 2011), may drive stability of aggressive behavior and its expression in different contexts, depending on developmental stage.

Low socioeconomic status (SES) may also partially explain continuity given that externalizing problems in childhood and adolescence – of which peer aggression is one expression – are linked to greater financial stress and lower social class in adulthood (Colman et al., 2009). Home environments that are low in socioeconomic status, in turn, pose a risk for aggressive behavior toward peers for offspring (e.g., Baker et al., 2020). Moreover, given the association between low SES and peer aggression and knowing that low SES is transmitted across generations as well, continuity in peer aggression might partly reflect continuity in low SES. Finally, socioeconomic disadvantages are sometimes associated with greater parenting problems (Madden et al., 2015), so it is feasible that both potential explanations for continuity in peer aggression across generations are interdependent.

### Moderators of Transmission

Variation in the strength of intergenerational transmission effects is feasible for different reasons and to capture for whom intergenerational transmission is stronger and who, by extension, is thus at higher risk for engaging in peer aggression if their parents have engaged in peer aggression as well, subgroup analyses that account for developmental period, offspring sex, and dyad composition are necessary but require larger samples than have been used in previous studies. To begin with, child peer aggression has partly different antecedents (e.g., Thomas et al., 2018) and outcomes than adolescent peer aggression (e.g., Sijtsema et al., 2009). Adolescent peer aggression is often assumed to be more transient (Moffit, 2006) and may thus be less predictive of offspring aggression and harsh parenting than childhood peer aggression. Within these developmental subgroups, offspring sex and parent-child dyad composition might moderate associations. That is, girls may be more strongly affected by parental influence compared to boys (e.g., Junttila & Vauras, 2009). That said, a mega-analysis concluded that the effects of family violence in children’s behavioral problems did not vary depending on sex of the children (Sternberg et al., 2006), suggesting that boys and girls are equally influenced by parents. Stronger effect on socialization processes may be present in same-sex parent-child dyads than opposite-sex dyads (Simpkins & Parke, 2001), although intergenerational transmission of criminal behavior was strongest in mother-daughter and weakest in father-son dyads (Besemer et al., 2017). In summary, various factors may influence the strength of associations and need to be considered as potential moderators.

## Current Study

To break continuity of negative peer behavior across generations and to better understand precursors to behavioral development, it is important to examine whether peer aggression is transmitted to the next generation and, if so, how. However, studies based on population samples and prospective reports where parent and offspring peer aggression was assessed during similar developmental periods are scarce. Retrospective reports might be biased and high-risk samples are not necessarily generalizable. To rectify the limitations of existing work, this study examined the intergenerational transmission of peer aggression using large, longitudinal multi-reporter samples. It was expected that child and adolescent peer aggression of parents (G1) would be transmitted to offspring (G2). Harsh parenting and SES were examined as potential explanations for intergenerational transmission, guided by the expectation that both would explain similarity in peer aggression across generations at least partly. Offspring sex and parent-child dyad composition were examined as moderators, both without directed hypotheses given ambiguous previous findings. Analyses were conducted separately for different developmental periods, with the expectation that childhood peer aggression would be more reflective of a general externalizing trait and thus more likely transmitted than peer aggression in adolescence.

## Methods

### Procedure and Participants

Data were used from the 1970 British Cohort Study (BCS70), which is an ongoing multi-disciplinary longitudinal study that monitors the development and lives of around 17,000 individuals in Britain. The mothers of 17,000 babies, born in a single week in April 1970, were asked if they would be willing to participate in the study with their newborn baby (Elliott & Shepherd, 2006). BCS70 has collected information about the health, educational, physical, and social development, and economic circumstances of participants. The initial BCS70 participants are referred to as Generation 1 (G1) in the present study and their offspring as Generation 2 (G2). For the analyses reported here, data from the following waves were used: 1975 (G1 age 5), 1980 (G1 age 10), 1986 (G1 age 16), and 2004 (G1 age 34). The overall participation rate for the waves used in this study was between 75.00% (G1 age 34) and 88.90% (G1 age 10). Detailed information on assessments and instruments used are provided in various cohort descriptions (Butler, Bynner, et al., 2016; Butler, Dowling, et al., 2016; Bynner et al., 2019; University of London et al., 2020).

During the first three waves, only data referring to G1 were collected. Information about G2 was collected when G1 participants were 34 years old, resulting in a wide age range of G2 because naturally G1 had started having children at different ages. To account for developmental differences among G2, different questionnaires were used to assess behavior of offspring aged 3 to 5 (G2 early childhood), 6 to 9 (G2 middle childhood), and 10 to 16 (G2 adolescence). By design, each G2 child belongs to only one age category.

In the first wave (G1 age 5), data were collected from 13,135 G1 participants. At G1 age 34, information about G2 was based on 5207 children from 2846 G1 participants. Only parent-child pairs were included in the analyses for whom information about peer aggression was available for both generations. Adopted children (*n* = 10) were excluded to account for genetic transmission. Some participants (G1) had more than one child (G2) in the same age category or had twins, which would introduce dependency of observations and violate modeling assumptions. Therefore, from these families, only the oldest G2 child was included in the analyses and one of the twins was selected randomly. Analyses are ultimately based on 2929 parent-child pairs.

The G1–G2 early childhood subsample contained 1132 G1 cohort members (59.8% female), mostly from the European ethnic group (40.5%). At age 34, most of G1 were married (79.4%), had a profession within the managerial-technical social class (30.1%), and had two children (54.9%). Children in the G1–G2 early childhood subsample had a mean age of 4.05 years.

The G1–G2 middle childhood subsample contained 1088 G1 cohort members (67.0% female), mostly from the European ethnic group (39.2%). At age 34, most of G1 in this subsample were married (72.2%), had a profession within the managerial-technical social class (25.1%) and had two children (56.7%). Children in the G1–G2 middle childhood subsample had a mean age of 7.52 years.

The G1–G2 adolescence subsample contained 709 G1 cohort members (78.3% female), mostly from the European ethnic group (35.4%). At age 34, most of G1 in this subsample were married (59.1%), had a profession within the skilled non-manual social class (19.6%) and had two children (46.4%). Children in the G1–G2 adolescence subsample had a mean age of 12.50.

### Measures

#### Peer aggression

G1 peer aggression was assessed at multiple time points during childhood and adolescence. All assessments were included to use these data as well as possible by forming latent variables with peer aggression assessments as indicators. To account for developmental differences in peer aggression, separate latent variables for early and middle childhood were created. Adolescent peer aggression was included as manifest variable.

In detail, G1 early childhood peer aggression was assessed at age 5 when parents reported on whether their child fought with other children or bullied others (1 = *doesn’t apply* to 3 = *certainly applies*). The responses to these two items were used as indicators for the G1 early childhood aggression latent variable. G1 middle childhood peer aggression was assessed at age 10 from parents who reported on whether their child fought with or bullied others (continuous, ranged from 0 *doesn’t apply* to 100 *certainly*). The responses to these two items were used as G1 middle childhood aggression indicators. G1 adolescence peer aggression was assessed at age 16 from parents who reported on whether their child bullied others (1 = *doesn’t apply* to 3 = *certainly applies*). This item was used as manifest variable. For G1 child and adolescent peer aggression measurements, no time frame was given.

G2 peer aggression was assessed from parents at the G1 age 34 assessment by asking parents whether their child had fought with or bullied other children over the past 6 months (1 = *not true* to 3 = *certainly true*). No age differences were made in the assessments.

#### Harsh parenting

Harsh parenting was assessed from parents who reported how often in the last 3 months they had told their child off, had shouted at their child, and had smacked their child on a 5-point Likert scale (1 = *never* to 5 = *daily*) at age 34. The responses to these items were used as three indicators for a latent variable representing harsh parenting.

#### Socioeconomic status

Social class of G1 at age 34 was used as an indicator of SES in the home environment of G2 (1 = *professional*, 2 = *Managerial-technical*, 3.1 = *Skilled non-manual*, 3.2 = *Skilled manual*, 4 = *Partly skilled*, 5 = *Unskilled*, 6 = *Other*). A previously published conceptualization by BCS70 researchers who use the British ‘Social Class based on occupation’ as indicator of SES was followed (see for example, Akasaki et al., 2019).

#### G2 sex

Sex of child was assessed from parents during the G1 age 34 assessment (1 = *male*, 2 = *female*). In the G2 early and middle childhood samples 52% were girls and in the G2 adolescence sample 49% were girls.

#### Parent-child dyad composition

Parent-child dyads were coded to represent father-son, father-daughter, mother-son, and mother-daughter dyads. In the G2 early childhood sample, 20% were father-son dyads and equally many were father-daughter dyads, 28% were mother-son, and 32% were mother-daughter dyads. In the G2 middle childhood sample, 18% were father-son dyads, 15% were father-daughter dyads, 34% were mother-son dyads, and 33% were mother-daughter dyads. Finally, in the G2 adolescence sample, 11% were father-son dyads and equally many were father-daughter dyads, 40% were mother-son dyads, and 38% were mother-daughter dyads.

### Analytic Strategy

R *psych* package version 1.9.1.31 (Revelle, 2019) was used for descriptive analyses and Hmisc package version 4.6-0 (Harrell, 2021) was used to create correlation heat maps. Because of the skewed distribution of peer aggression and harsh parenting, Spearman’s correlations were computed.

Analytic models were estimated as structural equation models in Mplus 8. In most models, G1 peer aggression was constructed as latent variable with two indicators except for G1 peer aggression in adolescence, which was assessed with one item only and this item was entered as manifest variable in the model. Generally, latent variables can be identified if three indicators are present, but all latent variables were estimated in a larger model, thus information could be “borrowed” from other items, allowing for a latent variable based on two indicators only (G1 early and middle childhood aggression). Measurement models and structural models were estimated simultaneously, in this way making use of all available information of each path (Loehlin & Beaujean, 2017). Harsh parenting as latent variable consisted of three items in all models. SES and G2 peer aggression consisted of single items in all models. Nine models were computed in total, these represent peer aggression assessed at different developmental periods in G1 and G2 (but note that one model did not converge). Each model consisted of direct effects from G1 to G2 peer aggressions as well as indirect effects via harsh parenting and via SES. Harsh parenting and SES were allowed to correlate in the model. Sex of G2 and dyad composition were included as covariates. Indirect effects were bootstrapped using 5000 draws.

Bootstrapping required the use of maximum likelihood estimation, missing data were accounted for by using full information maximum likelihood estimation. For each model, *X*^*2*^, Comparative Fit Index (CFI), Tucker-Lewis Index (TLI), Standardized Root Mean Square Residual (SRMR), and Root Mean Square Error of Approximation (RMSEA) were evaluated. Models with a nonsignificant *X*^*2*^, i.e., *p* > 0.05 (Barrett, 2007), a CFI value larger than 0.90, a SRMR value lower than 0.10 (Kline, 2005), and a TLI value close to 1.0 (Loehlin & Beaujean, 2017) were considered well-fitting. For RMSEA, a value less than 0.06, was considered to constitute a good fit (Hu & Bentler, 1999).

To test whether effects differed by G2 sex or dyad composition, models were also computed in a multiple group framework, to evaluate whether model fit improved significantly (evaluated as Chi2 difference) when girls and boys, and different dyad compositions (mother-son, mother-daughter, father-son, father-daughter) were free to vary in substantive associations between variables. Given the large number of conducted comparisons (nine per potential moderator), a *p* value threshold of *p* < 0.01 was handled as cut-off for further interpretation.

## Results

### Descriptive Statistics

**Fig. 1:**
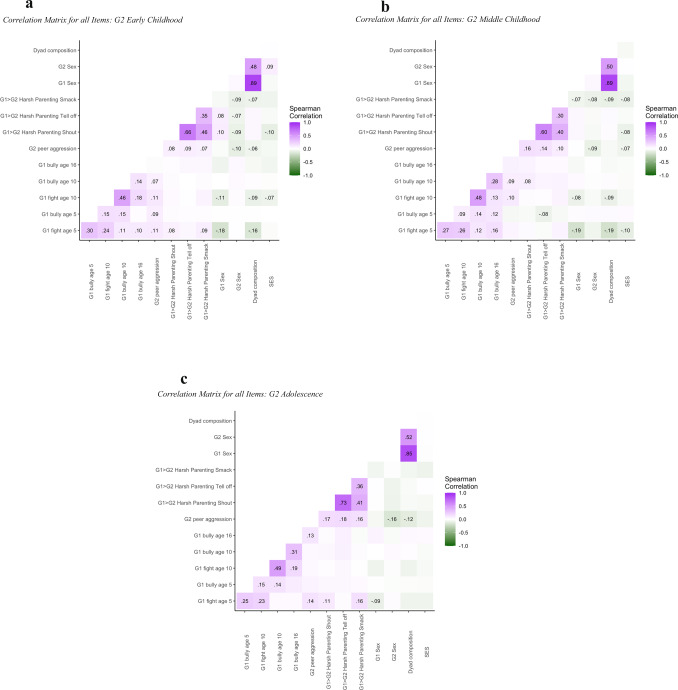
**a**−**c** Correlation Matrices for all Items for G2 Early Childhood, G2 Middle Childhood, and G2 Adolescence. *Note*. Correlation coefficients are presented for significant correlations only

Descriptive statistics for the peer aggression, harsh parenting, and SES variables are presented separately by subsample in Table 1 and bivariate correlations can be found in Fig. 1a–c. Note that descriptive statistics and correlations are based on complete data whereas subsequent models make use of full information maximum likelihood estimation. Parent peer aggression was partly associated with offspring peer aggression, with most associations found for the G2 early childhood subsample. Parent peer aggression and later harsh parenting were hardly associated whereas harsh parenting was linked to offspring peer aggression in all samples and for all aspects of harsh parenting.

**Table 1:** Descriptive Statistics of G1 and G2 Peer Aggression and G1 Harsh Parenting

	G2 early childhood (*n* = 1132)				G2 middle childhood (*n* = 1088)				G2 adolescence (*n* = 709)			
	*n* (%)	Mean	SD	Range given answers	*n* (%)	Mean	SD	Range given answers	*n* (%)	Mean	SD	Range given answers
**G1 early childhood peer aggression**												
Fights with children (age 5, PR)	943 (83.30)	1.31	0.50	1–3	905 (83.18)	1.35	0.53	1–3	569 (80.25)	1.40	0.57	1–3
Bullies children (age 5, PR)	942 (83.22)	1.12	0.35	1–3	902 (82.90)	1.14	0.37	1–3	569 (80.25)	1.17	0.41	1–3
**G1 middle childhood peer aggression**												
Fights with children (age 10, PR)	993 (87.72)	16.81	18.61	0–96	956 (87.87)	16.78	18.82	0–96	589 (83.07)	18.50	20.42	0–97
Bullies children (age 10, PR)	993 (87.72)	13.96	14.45	0–97	956 (87.87)	13.81	14.73	0–99	590 (83.22)	14.57	15.55	0–97
**G1 adolescence peer aggression**												
Bullies others (age 16, PR)	726 (64.13)	1.06	0.27	1–3	693 (63.69)	1.08	0.30	1–3	435 (61.35)	1.08	0.32	1–3
**G2 peer aggression**												
Fights or bullies children (PR)	1132 (100)	1.10	0.32	1–3	1088 (100)	1.11	0.35	1–3	691 (97.50)	1.18	0.46	1–3
**G1 harsh parenting**												
Shouted at your child (PR)	1130 (99.82)	3.47	0.99	1–5	1084 (99.63)	3.36	1.00	1–5	692 (97.60)	3.19	1.02	1–5
Told your child off (PR)	1126 (99.47)	3.86	0.93	2–5	1083 (99.54)	3.61	0.97	1–5	686 (96.76)	3.33	0.99	1–5
Smacked your child (PR)	1131 (99.91)	1.87	0.83	1–5	1085 (99.72)	1.74	0.71	1–5	691 (97.46)	1.53	0.62	1–5
**G1 SES**												
Social class (PR)	879 (77.65)	2.74	0.89	1–6	827 (76.01)	2.92	0.86	1–6	519 (73.20)	3.16	0.85	1–6

G2 early childhood = age 3–5; G2 middle childhood = age 6–9; G2 adolescence = age 10–16; *PR* parent-report

### Is Peer Aggression Transmitted Across Generations?

**Fig. 2:**
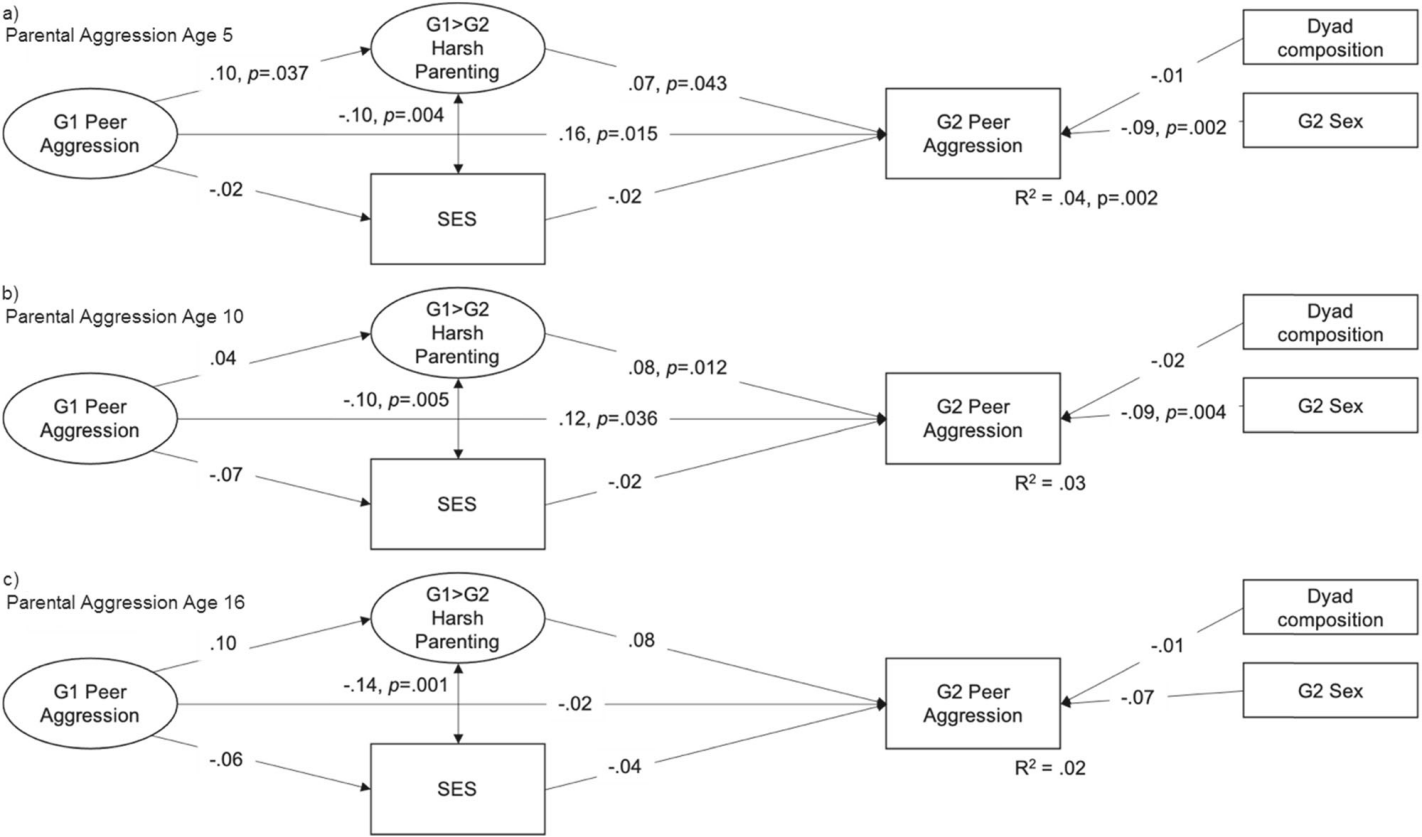
Associations Between G1 Peer Aggression, Harsh Parenting, SES, and G2 Peer Aggression in Early Childhood. *Note*. G2 early childhood = age 3–5; *P* values are presented for significant associations, model fit was satisfactory to good with RMSEA < 0.06, CFI > 0.95 and srmr < 0.04

**Fig. 3:**
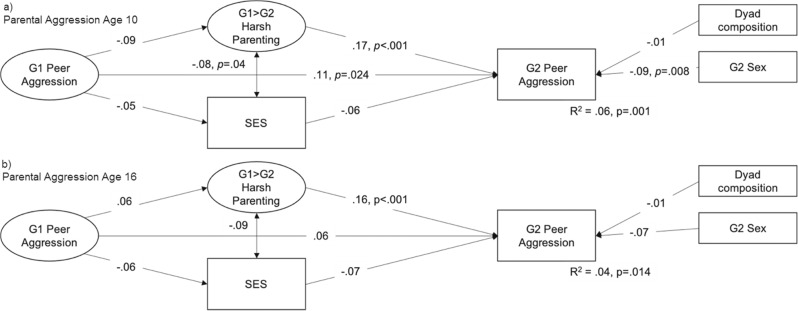
Associations Between G1 Peer Aggression, Harsh Parenting, SES, and G2 Peer Aaggression in middle childhood. *Note.* G2 middle childhood = age 6–9; *P* values are presented for significant associations, model fit was satisfactory to good with RMSEA < 0.06, CFI > 0.95 and srmr < 0.04; Note that a model in which G1 peer aggression was assessed at age 5 was also estimated, but this model did not converge

**Fig. 4:**
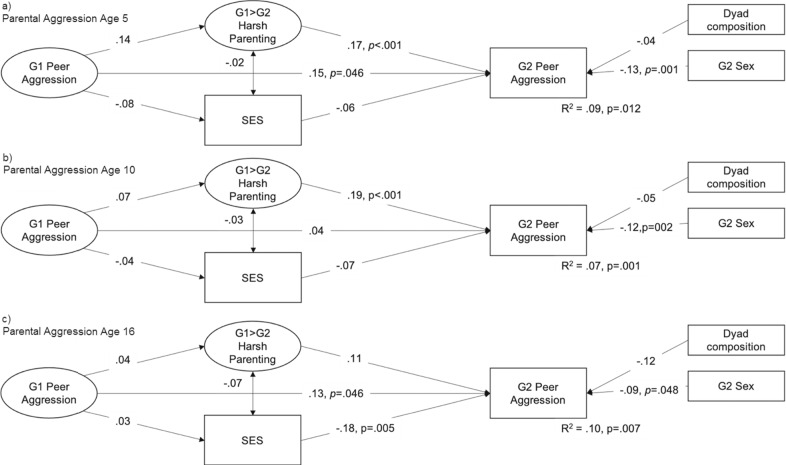
Associations Between G1 Peer Aggression, Harsh Parenting, SES, and G2 Peer Aggression in Adolescence. *Note*. G2 adolescence = age 10–16; *P* values are presented for significant associations, model fit was satisfactory to good with RMSEA < 0.06, CFI > 0.95 and srmr < 0.04

Figures 2–4 depict core elements of the computed structural equation models to examine associations between parents’ and offspring peer aggression. Note that to keep figures concise and readable, factor loadings are not presented, these were all > 0.30. One model did not converge (peer aggression in parent early childhood as predictor of offspring middle childhood peer aggression). Results for the remaining eight models are mixed with respect to direct associations between parent and offspring aggression: Whereas associations between parent and offspring peer aggression as assessed in similar developmental periods were always significantly related, no associations between parent peer aggression in adolescence and offspring peer aggression in early and middle childhood were found. No association between parent peer aggression in middle childhood and offspring peer aggression in adolescence was found.

### Are Harsh Parenting and SES Transmission Mechanisms?

Also included in the models presented in Figs. 2–4 are indirect effects via harsh parenting and SES. Whereas harsh parenting was associated with offspring peer aggression in six out of eight models, parent peer aggression was not linked to their own harsh parenting later on, with one exception: G1 early childhood peer aggression predicted G1 harsh parenting. In most models, SES was not associated with parent or offspring peer aggression. None of the formal tests of indirect effects were significant. This means that the data used here provide no support that harsh parenting or SES explain the intergenerational transmission of peer aggression.

### Do Effects Differ by Offspring Sex and Parent-Child Dyad Composition?

Multiple group models were computed for offspring sex (boys versus girls) and dyad composition (father-son, father-daughter, mother-son, mother-daughter). When offspring sex was tested as moderator, a significant difference in fit was found for one model (out of eight). Specifically, the association between parent peer aggression in middle childhood and offspring peer aggression in early childhood was larger for boys (*β* = 0.20, *p* = 0.04) than for girls (*β* = 0.05, *p* = 0.46). All other substantive paths were of similar magnitude for boys and girls in this model. One of the eight models did not converge, and moderation by offspring sex for the model in which parent peer aggression in early childhood predicts offspring peer aggression in middle childhood was not explored because this model had already not converged in initial analyses. Effects could thus be constrained to be equal across boys and girls in six models, suggesting limited support for offspring sex as moderator. When dyad composition was tested as moderator, modeling issues were common, resulting in non-convergence of three unconstrained models, specifically those involving parental aggression in middle childhood and warnings pertaining to small group sizes. These comparisons need to be interpreted with caution but did not suggest any differences in effect sizes as a function of parent-child dyad composition.

## Discussion

A plethora of studies have examined risk factors for child and adolescent aggressive behavior toward peers but only few have considered the role of parents’ developmental histories. This is a shortcoming of the peer aggression literature, given empirical support for intergenerational transmission of other forms of aggressive behavior. To gain a comprehensive understanding of the origins of peer aggression and to rigorously test whether peer aggression shows continuity across generations, an intergenerational perspective is needed. For this, data collected in both generations in similar developmental periods are most suited. Fortunately, the BCS70 study has followed participants at regular intervals across childhood and adolescence and into adulthood and also includes information on offspring. The available data on peer aggression for both generations makes BCS70 suitable for rigorous examination of intergenerational continuity without relying on retrospective reports or data from vastly different developmental periods. As hypothesized, parents peer aggression was linked to offspring peer aggression in most models, suggesting support for a continuity across generations. In contrast to expectations, neither harsh parenting nor SES explained the peer aggression transmission. The strength of the transmission did not vary for offspring sex nor among parent-child dyad compositions.

### Transmission of Peer Aggression Across Generations

In detail, parents’ peer aggression was associated with offspring peer aggression in five of the nine models. Continuity of peer aggression was found when peer aggression of parents and children were assessed in similar developmental periods. This pattern of findings is in line with the suggestion that the social behavior of one generation in a certain developmental period is particularly comparable to the social behavior of the next generation in the similar developmental period (Conger et al., 2003). Although the findings provide some support for intergenerational transmission of peer aggression, the effects are weaker than, for instance, for transmission of harsh parenting (e.g., Kerr et al., 2009), intimate partner violence (Shakoor et al., 2020), and crime (e.g., Besemer et al., 2017). Larger effect sizes may emerge in less specific models, i.e., when broad-band aggression or behaviors that are more encompassing are examined. A criminal lifestyle, for instance, is reflected across life domains and violent relationships can be expressed in various negative interactions including shouting, ignoring, physical violence, and fighting, across different situations. Peer aggression, in contrast, is possibly more restricted to particular behaviors – at least those that were assessed in the present study – and contexts. As such, peer aggression as a narrower, more specific concept may not have such an effect on the next generation as crime or intimate partner violence.

In general, peer aggression may be more likely to be transmitted to the next generation if it is stable and persistent throughout childhood and adolescence, rather than fleeting and temporary. As such, context conditions that add to stability such as a classroom climate with aggression-friendly norms is likely more conducive to longevity of aggression (Dijkstra et al., 2008) and, eventually, to its transmission, than a context where peer aggression is negatively evaluated and punished (Jackson et al., 2015). In this initial study no contextual factors such as classroom norms were explored but future research with information about norms within the societal context may help elucidate why transmission is sometimes present and at other times not. What is more, to capture the role of stability in peer aggression as increasing or decreasing the risk for intergenerational continuity requires repeatedly measured aggression. Multiple assessments of G1 peer aggression over time were included in the BCS70 data, but because these differed in content, development over time could not be modeled. As rigorously assessed cohorts of children and adolescents such as the Tracking Adolescents’ Individual Lives Survey, the Australian Temperament Project, and the Avon Longitudinal Study of Parents and Children grow up and spin-off next generation studies are introduced, advanced developmental modeling of behavior in G1 and effects of behavioral stability on G2 will become options for future research.

### Mechanisms of Transmission

Harsh parenting and SES were tested as transmission mechanisms, yet no convincing support for these transmission pathways was found. In contrast to prior work, harsh parenting was not a correlate of own earlier peer aggression (Dubow et al., 2003). It is possible that stability of peer aggression not only plays a role for transmission, but also for whether or not aggressive behavior in childhood and adolescence is linked to own parenting. Harsh parenting was associated with offspring peer aggression in most models, which is in line with previous studies (e.g., Conger et al., 2003) and the concept of spillover where parent-child relationships can shape emotions and behaviors that affect social interactions in the peer context and vice versa (Kaufman et al., 2020). Harsh parenting and offspring peer aggression may thus mutually influence each other. Of note, harsh parenting as well as G2 peer aggression are reported on by the same person, which might have confounded this link. Interestingly, SES was not associated with parents’ peer aggression nor with offspring’s peer aggression, in contrast to many studies that found such links (e.g., Baker et al., 2020). It is possible that parents’ educational attainment and household income play a more important role than occupational status that was used as a proxy for SES in the current study.

Whereas harsh parenting and SES were tested in the current study as transmission mechanisms, genetic mediation is just as, if not more, likely. Specifically, parents’ genes that explain part of the variance in G1 peer aggression are transmitted to offspring and explain then part of the variance in G2 peer aggression. What is more, the same genes that explain G1 peer aggression might also explain variance in G1 harsh parenting. Rigorous tests of genetic transmission that combine the benefits of the current study – assessment of child behavior at approximately the same age rather than using retrospective reports – with the inclusion of multiple reporters’ perspectives and genetic information are rare but innovative genetically-informed multiple-generation studies (Kretschmer, 2021) will play an important role in allowing for such research.

### Limitations and Future Directions

Despite the insights gained, the results of this study should be interpreted with some limitations in mind. To begin with, only the transmission of peer aggression from one parent to offspring was examined and, in this sense, the influence of the other parent in the transmission process has been neglected. Yet, the role of one parent’s behavior and experiences in predicting behavior problems in offspring cannot be fully understood without being mindful of the other parent’s influence as they may act as buffer or risk factor (Jeon & Neppl, 2019). Information about both parents and their developmental history is thus needed to comprehensively study intergenerational transmission of peer-related behavior and experiences.

Next, even with the advantage that peer aggression was measured in both childhood and adolescence for two generations, one might still wonder whether the same behavior across generations was examined. The assessments for parents’ peer aggression took mostly place during the early 1970s to the mid-1980s. Peer aggressive behavior in that time may be perceived in a different light compared to the peer aggressive behaviors of their offspring almost 30 years later, especially considering that the political, social, and academic attention on peer aggressive behaviors has surged during the last couple of decades (Smith, 2004). It is likely that the increased attention for peer aggression, starting from the beginning of the 1990s, has affected the perception of peer aggression and this may also apply to the assessments used for offspring peer aggression or how parents report offspring aggression. In general, intergenerational studies that use assessments from similar developmental periods include long time-spans and changes in interpretation and perception of concepts may change and macro-level variables may affect continuity. In fact, any longitudinal study that seeks to examine continuity of constructs can encounter this and related problems such as outdatedness of instruments. However, the benefits of such studies that avoid retrospective bias and allow for studying long-term development or transmission of a particular behavior or experience during the same developmental phase outweigh these limitations.

Further, aggression towards peers can take different forms, e.g., physical, relational, or electronic, and it is possible that transmission across generations depends on the form of peer aggression. In the present study, predominantly physical peer aggression was examined. Effect sizes for intergenerational transmission may be even weaker for other forms of peer aggression. To speculate, it may be that overt and physical aggression toward others are more easily observed by offspring and thus have a greater impact on offspring behavior. Moreover, exerting physical aggression toward others is not normative and usually societally disapproved of. If G1 has engaged in such behavior, this may indicate a more overarching lack of acceptance of common norms, which, in turn, may have an impact on family life and, eventually, influence norms and behavior in G2. In other words, physically aggressive behavior may be indicative of a generally more antisocial lifestyle, which would then explain stronger transmission. Of note, parent assessments of peer aggression were done in the 1970s and 80s, before electronic forms of aggression were possible. This does not mean that parents’ engagement in physical or relational aggression could not function as a potential predictor for offspring electronic aggression. Future research into whether some forms of peer aggression show greater intergenerational continuity than others would be beneficial.

Finally, the findings are based on parental reports only, which means that the perspectives of peers and teachers are lacking. Parents have information about aggressive behavior at home but may be missing insight into behaviors in the peer context, as peer aggression naturally occurs among peers – often at school. Peer nominations are gold standard nowadays (Marks, 2017) but were not part of standard research practices in the 1970s, at which time the assessments used took place, certainly not in birth cohorts of several thousand participants. Perspectives of teachers may also complement those of parents. Yet, teacher reports on peer aggression were only available for parents’ peer aggression at age 10, which means that it was not possible to derive comparable measures from teacher reports and only parents’ perspectives could be included.

## Conclusion

To break chains of negative peer behavior across generations, it is important to understand why some children and adolescents aggress against their peers and how parents’ developmental histories play a role in this. The aim of the present study was therefore to examine whether peer aggression is transmitted to the next generation and, if so, via which mechanisms. In most models, parents’ peer aggression was associated with offspring peer aggression – especially for similar developmental stages – suggesting some degree of continuity across generations. Neither harsh parenting nor SES explained the continuity of peer aggression. Studies focusing on the effects of stable peer aggression and contextual social norms may help elucidate why transmission is sometimes present and at other times not. Future research would also benefit from including information on peer-related experiences of both parents, as well as genetic information to understand their role in intergenerational transmission.
